# Retrospective study of chidamide-containing regimens as maintenance therapy among T- and NK-cell lymphoma patients

**DOI:** 10.3389/fonc.2025.1507418

**Published:** 2025-03-03

**Authors:** Tao Hai, Wanchun Wu, Kexin Ren, Na Li, Liqun Zou

**Affiliations:** Division of Medical Oncology, Cancer Center and State Key Laboratory of Biotherapy, Sichuan University West China Hospital, Chengdu, China

**Keywords:** chidamide, T and NK-cell lymphoma, maintenance therapy, survival, safety

## Abstract

**Objective:**

This study aimed to explore the efficacy of chidamide-containing regimens as maintenance therapy in patients with T- and natural killer (NK)-cell lymphomas (TNKLs).

**Methods:**

A total of 51 patients with TNKLs who received chidamide-containing regimens after induction therapy were enrolled. The primary end point was progression-free survival (PFS), while the secondary end point was overall survival (OS) and safety.

**Results:**

The median duration of maintenance was 14 months (range = 1–24 months). Most of the patients were diagnosed with extranodal NK/T-cell lymphoma (ENKTCL; 24/51, 47.1%), followed by angioimmunoblastic T-cell lymphoma (AITL; 14/51, 27.5%). The median PFS and OS were 21 and 29 months, respectively. The 2-year PFS and OS among the overall population were 45.1% and 54.2%, respectively. Patients who experienced complete remission (CR) after induction therapy had favorable survival compared with non-CR patients (partial remission/stable disease, PR/SD). Patients who experienced CR after first-line induction treatment also had favorable survival, but similar significance was not observed in the salvage treatment group. Although 86.3% of the patients had chidamide-related adverse events (AEs), severe hematological AEs (grade ≥3) occurred in only 11 (21.6%) patients, indicating the safe toxicity profile of chidamide.

**Conclusion:**

The prolonged survival indicated that chidamide-containing maintenance therapy is promising and well tolerated in patients with TNKLs.

## Introduction

The incidence of T-cell and natural killer (NK)-cell lymphomas (TNKLs) is less than one case per 100,000 people in the United States based on SEER registries (1). The most frequent subtype is PTCL-NOS (peripheral T-cell lymphoma not otherwise specified), which accounted for 25.9%, followed by angioimmunoblastic T-cell lymphoma (AITL; 18.5%), anaplastic large-cell lymphoma (ALCL; 12%), and extranodal NK/T-cell lymphoma (ENKTCL; 10.4%). However, there are regional differences in frequency among the common subtypes. PTCL-NOS is more common in North America than in European and Asian countries, while ENKTCL and AITL are more common in Asia (2).

Most patients with TNKLs are treated with chemotherapy [asparaginase-containing regimen for NK/T-cell lymphoma, but CHOP (cyclophosphamide/doxorubicin/vincristine/prednisone) or a CHOP-like regimen for others]. A retrospective meta-analysis showed a pooled 3-year overall survival (OS) of 49%–61% among patients with TNKLs treated with anthracycline-based regimens who demonstrated poor prognosis (3, 4). Patients who experienced refractory or relapsed disease had an extremely poor OS of 5.8 months (5). Although new agents or regimens have been approved in recent years, there is an urgent need for effective drugs after response to first-line or salvage treatment.

The dynamic expression of histone deacetylase (HDAC) and acetyltransferase controls the normal structure of chromatin and the gene transcription, and it could be frequently detected in hematological malignancies. HDAC is essential for the regulation of gene transcription and various signaling pathways (6). The HDAC inhibitors romidepsin and belinostat have been approved by the US Food and Drug Administration (FDA) for the treatment of refractory and relapsed PTCLs (7, 8). Similarly, the newly developed HDAC inhibitor chidamide, which was designed to selectively target HDAC1–3 and HDAC10, was approved by the Chinese Food and Drug Administration (CFDA) based on the results of a pivotal phase II trial in refractory and relapsed PTCLs (9). Several studies have demonstrated that chidamide combined with chemotherapy exhibited an improved progression-free survival (PFS) or overall survival (OS) in first-line treatment (10, 11). The aim of this retrospective study was to reveal the benefit of chidamide combined with chemotherapy and maintenance treatment in patients with TNKLs.

## Materials and methods

### Study design and patients

This is a retrospective, open-label study of a chidamide-containing comprehensive treatment among patients with TNKLs. Patients were required to be 18 years or older and assessed to have an Eastern Cooperative Oncology Group (ECOG) performance score of 0 or 1. Patients were strictly confirmed by senior pathologists to have a diagnosis of the TNKL subtypes in accordance with the WHO 2016 classification of lymphoid neoplasms, including PTCL-NOS, ENKTCL, AITL, ALCL, MF (mycosis fungoides), and MEITL (monomorphic epitheliotropic intestinal T-cell lymphoma), and staged according to the Ann Arbor staging criteria. The other eligibility criteria included no apparent evidence of other malignancies and the availability of complete clinical data. This study was approved by the Research Ethics Committee of the West China Hospital.

The clinicopathological characteristics included age, sex, pathological subtypes, Ann Arbor stage, International Prognostic Index/Prognostic Index of Natural Killer Lymphoma/Epstein–Barr Virus (IPI/PINK-E) classified into low-risk and non-low risk, induction treatment, and response evaluation, among others. The data for all aforementioned variables were used in the analysis.

### Treatment and procedures

Patients who achieved complete remission (CR), partial remission (PR), or stable disease (SD) continued to receive chidamide as a maintenance treatment after induction therapy (first-line or salvage treatment). For maintenance treatment, patients were treated with chidamide 30 mg two times a week (with a one-third decrease in dosage if other drugs were used or severe toxicity emerged). Chidamide was combined with the induction chemotherapy regimens (as a first-line or salvage treatment) during cycle 1 or cycle 2 as an early intervention (EI). Initial assessment was conducted after four cycles of treatment with positron emission tomography/computed tomography (PET/CT) using the International Work Group (IWC) criteria (12). For patients diagnosed with ENKTCL who were treated with immune checkpoint inhibitor (ICI)-containing regimens, EI was not required.

### Maintenance treatment

The maintenance treatment regimens were determined by induction chemotherapy. Chidamide was combined with ICIs or was administered alone for ENKTCL, chidamide was combined with immunomodulatory drugs (e.g., lenalidomide or thalidomide) or was administered alone for AITL and MF, while chidamide alone was administered for PTCL-NOS, ALCL, and MEITL. Regimens of chidamide only or chidamide in combination with other drugs were defined as chidamide (C−) or chidamide plus (C+), respectively. The entire maintenance procedure was designed for up to 2 years, unless disease progression or unacceptable toxicity occurs.

### End point and safety

The primary end point was PFS, which was determined as the time period between the initial chidamide reception and disease recurrence, disease progression, last follow-up, or death. The secondary end point was OS, which was determined as the time period from the initial diagnosis to the last follow-up or death from any cause, as well as safety. The dynamics of the laboratory parameter monitoring and physical examination were included in the safety assessment. Adverse events (AEs) were graded using the National Cancer Institute Common Toxicity Criteria for Adverse Events Scale, version 4.0.

### Statistical analysis

Continuous variables are presented as median and range, while categorical variables are presented as frequency with percentage. OS and PFS were described using Kaplan–Meier survival curves. All statistical analyses were performed using SPSS software (version 29.0.1.0). The graphics and tables were plotted using R software (version 4.2.2) and Microsoft Office software.

## Results

**Figure 1:**
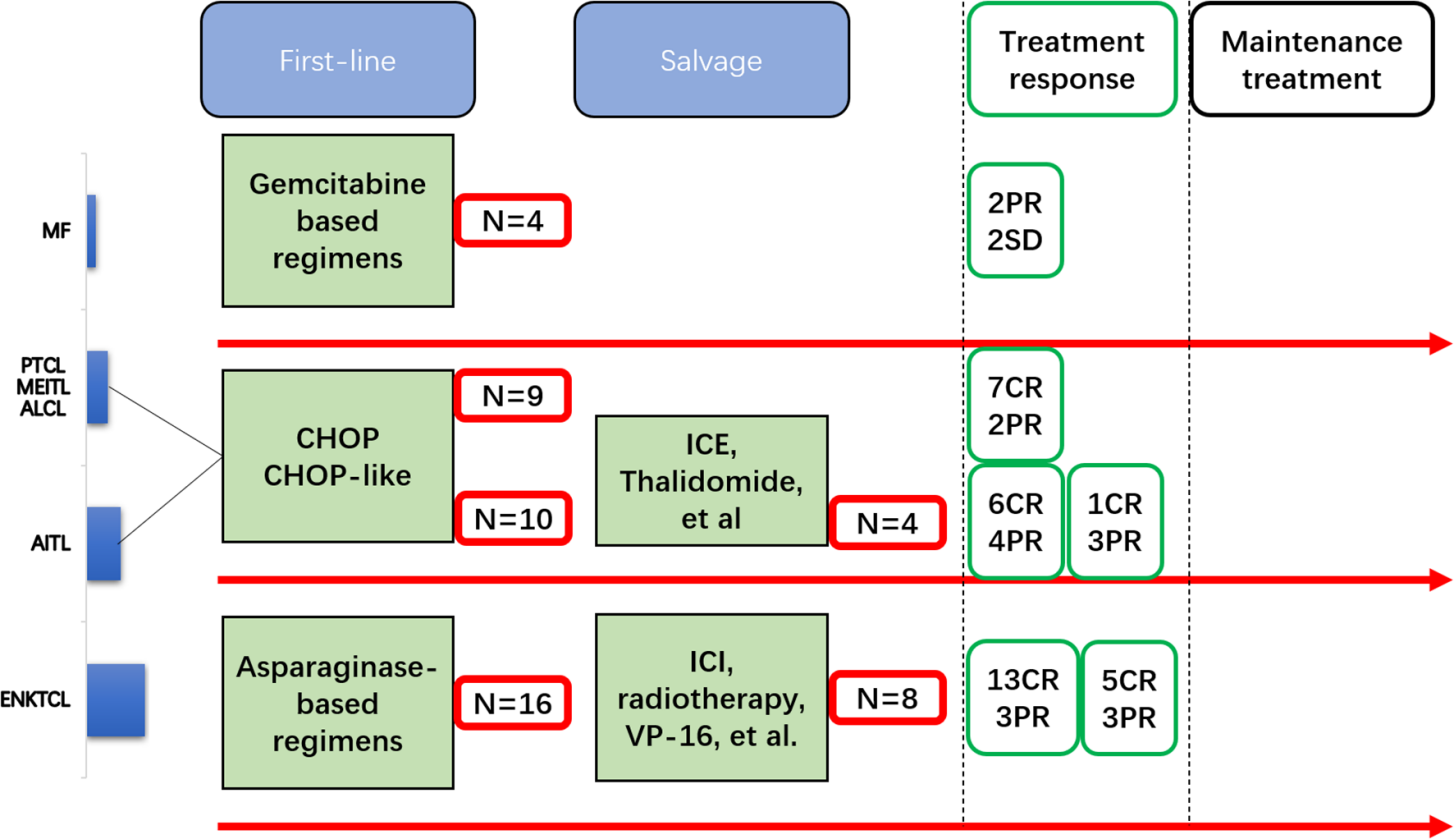
Flowchart of the patients with T- and natural killer (NK)-cell lymphomas (TNKLs) undergoing induction therapy to maintenance therapy.

A total of 51 patients were enrolled in this study from January 2018 to September 2024 at the West China Hospital (Chengdu, China) (**Figure 1**). The baseline characteristics of the enrolled patients are shown in **Table 1**. The median age of the overall population was 73 years (range = 15–81 years). Most of the patients were diagnosed with ENKTCL (24/51, 47.1%), followed by AITL (14/51, 27.5%), and then other subtypes including PTCL-NOS (4/51, 7.8%), MF (4/51, 7.8%), ALCL (3/51, 5.9%), and MEITL (2/51, 3.9%). There were 22 patients who were diagnosed as stage I/II, while 18 (35.3%) patients were considered low risk (IPI = 0–1 or PINK-E = 0–1).

**Table 1:** Baseline characteristics of the enrolled patients.

Characteristics	Overall	ENKTCL	AITL	PTCL-NOS	MF	ALCL	MEITL
	*N* = 51 (%)	*n* = 24 (%)	*n* = 14 (%)	*n* = 4 (%)	*n* = 4 (%)	*n* = 3 (%)	*n* = 2 (%)
Sex							
Male	32 (62.7)	7 (29.2)	11 (78.6)	1 (25.0)	1 (25.0)	0 (0)	2 (100)
Female	19 (37.3)	17 (70.8)	3 (21.4)	3 (75.0)	3 (75.0)	3 (100)	0 (0)
Age (years)							
Median (range)	73 (15–81)	48 (15–73)	(53–78)	66 (64–81)	56 (42–67)	60 (49–70)	NA
Stage							
I/II	22 (43.1)	14 (58.3)	5 (35.7)	1 (25.0)	0 (0)	1 (33.3)	1 (50.0)
III/IV	29 (56.9)	10 (41.7)	9 (64.3)	3 (75.0)	4 (100)	2 (66.7)	1 (50.0)
IPI/PINK-E							
Low risk	18 (35.3)	10 (41.7)	6 (42.9)	1 (25.0)	0 (0)	1 (33.3)	0 (0)
Non-low risk	33 (64.7)	14 (58.3)	8 (57.1)	3 (75.0)	4 (100)	2 (66.7)	2 (100)
Induction therapy							
First line	39 (76.5)	16 (66.7)	10 (71.4)	4 (100)	4 (100)	3 (100)	2 (100)
Salvage	12 (23.5)	8 (33.3)	4 (28.6)	0 (0)	0 (0)	0 (0)	0 (0)
Baseline responses							
CR	32 (62.7)	18 (75.0)	7 (50.0)	3 (75.0)	0 (0)	2 (66.7)	2 (100)
Non-CR (PR/SD)	19 (37.3)	6 (25.0)	7 (50.0)	1 (25.0)	4 (100)	1 (33.3)	0 (0)

*ENKTCL*, extranodal natural killer/T-cell lymphoma; *AITL*, angioimmunoblastic T-cell lymphoma; *PTCL-NOS*, peripheral T-cell lymphoma, not otherwise specified; *MF*, mycosis fungoides; *ALCL*, anaplastic large-cell lymphoma; *MEITL*, monomorphic epitheliotropic intestinal T-cell lymphoma; *IPI*, International Prognostic Index; *PINK-E*, Prognostic Index of Natural Killer Lymphoma/Epstein–Barr Virus; *CR*, complete remission; *PR*, partial remission; *SD*, stable disease; *NA*, not available.

All patients with ENKTCL received asparaginase-based regimens [e.g., vincristine/daunorubicin/l-asparaginase/prednisone (VDLP) and gemcitabine/l-asparaginase/ifosfamide/dexamethasone/etoposide (GLIDE)] as a first-line treatment. Those with the remaining subtypes received CHOP or CHOP-like regimens as a first-line treatment, with the exception of patients with MF who received gemcitabine-based regimens.

Before the maintenance procedure started, 32 (62.7%) patients had CR after induction therapy (first-line or salvage), whereas 39 (76.5%) patients showed response (26 CR and 13 non-CR) after the first-line treatment. A total of 22 (43.1%) patients received oral chidamide in combination with induction therapy (as EI) and then transferred to the maintenance procedure. The median duration of maintenance therapy (DOM) was 14 months (range = 1–24 months).

In total, the median PFS and OS were 21 and 29 months, respectively. The 2-year PFS and OS in the overall population were 45.1% and 54.2%, respectively. Due to the lack of data, the survival analysis for patients with PTCL-NOS, MF, ALCL, and MEITL was not presented.

**Figure 2:**
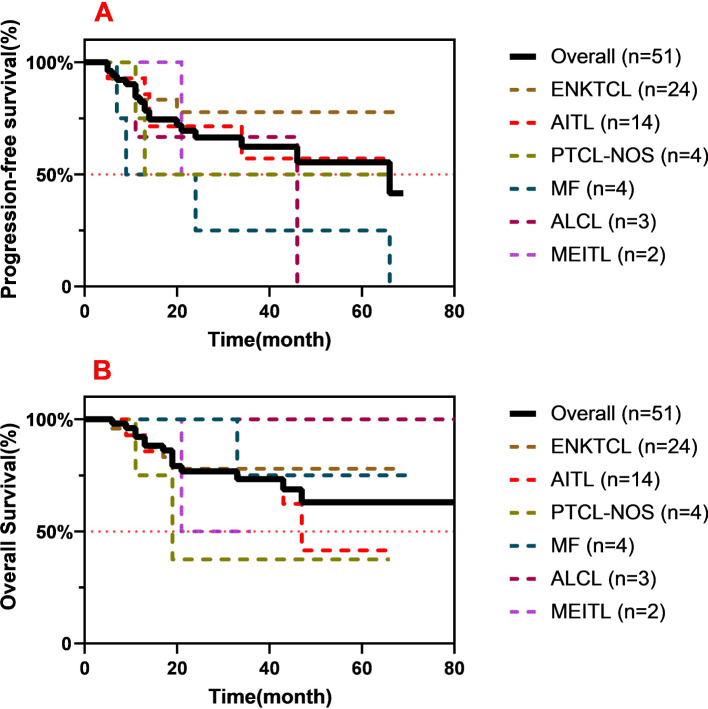
Progression-free survival (PFS) **(A)** and overall survival (OS) **(B)** among all subtypes of T- and natural killer (NK)-cell lymphomas (TNKLs).

For patients diagnosed with ENKTCL and AITL, the 2-year PFS rates were 57.1% and 56.9%, respectively (**Figures 2A, B**). Regardless of the treatment lines, the patients with ENKTCL had much higher rates of CR (first-line, 81.3%; salvage, 62.5%) compared with the patients with AITL. However, the patients with AITL had a favorable 2-year OS (64.3% *vs*. 58.3%). The neoadjuvant response index (NRI) model for the patients with ENKTCL was as follows: >60 years old (6, 25.0%), stage III/IV (10, 41.7%), increased lactate dehydrogenase (LDH) (12, 50.0%), ECOG 0–1 (22, 91.7%), and primary tumor invasion (PTI) (13, 54.2%).

**Figure 3:**
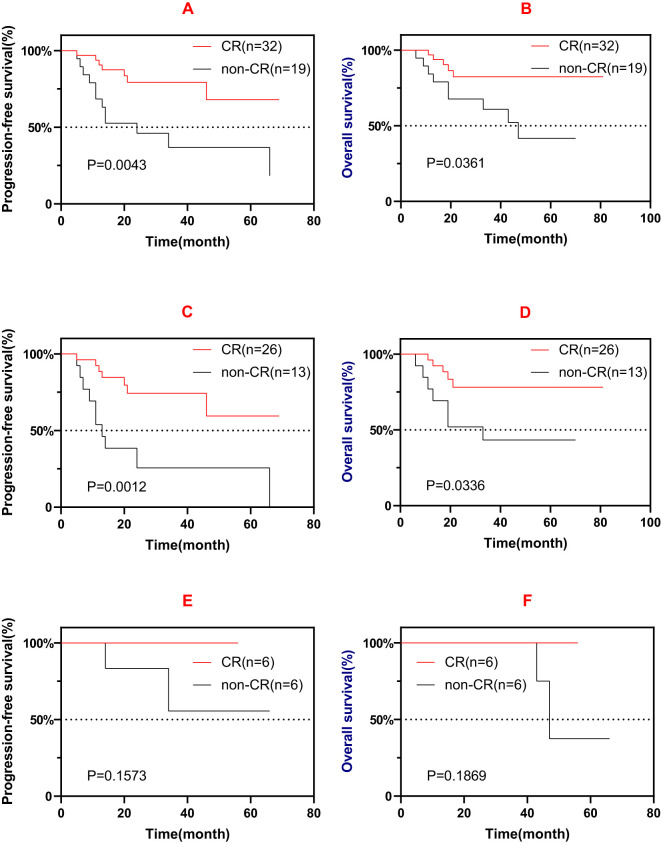
**(A, B)** Progression-free survival (PFS) **(A)** and overall survival (OS) **(B)** of patients who experienced complete remission (CR) and those non-CR after induction therapy (first-line or salvage). **(C, D)** PFS **(C)** and OS **(D)** of patients who experienced CR and those non-CR after first-line induction therapy. **(E, F)** PFS **(E)** and OS **(F)** of patients who experienced CR and those non-CR after salvage induction therapy.

For the patients with EI and those without EI, the results of the survival analysis were not significant (PFS: *p* = 0.772; OS: *p* = 0.973). For the patients with C+ and C−, the results of the survival analysis were also not significant (PFS: *p* = 0.798; OS: *p* = 0.352). Patients who experienced CR after induction therapy had favorable survival compared with non-CR (PR/SD) patients (**Figures 3A, B**). Furthermore, those who experienced CR after the first-line induction treatment also had favorable survival (**Figures 3C, D**). However, similar significance was not observed in the salvage treatment group (**Figures 3E, F**).

**Figure 4:**
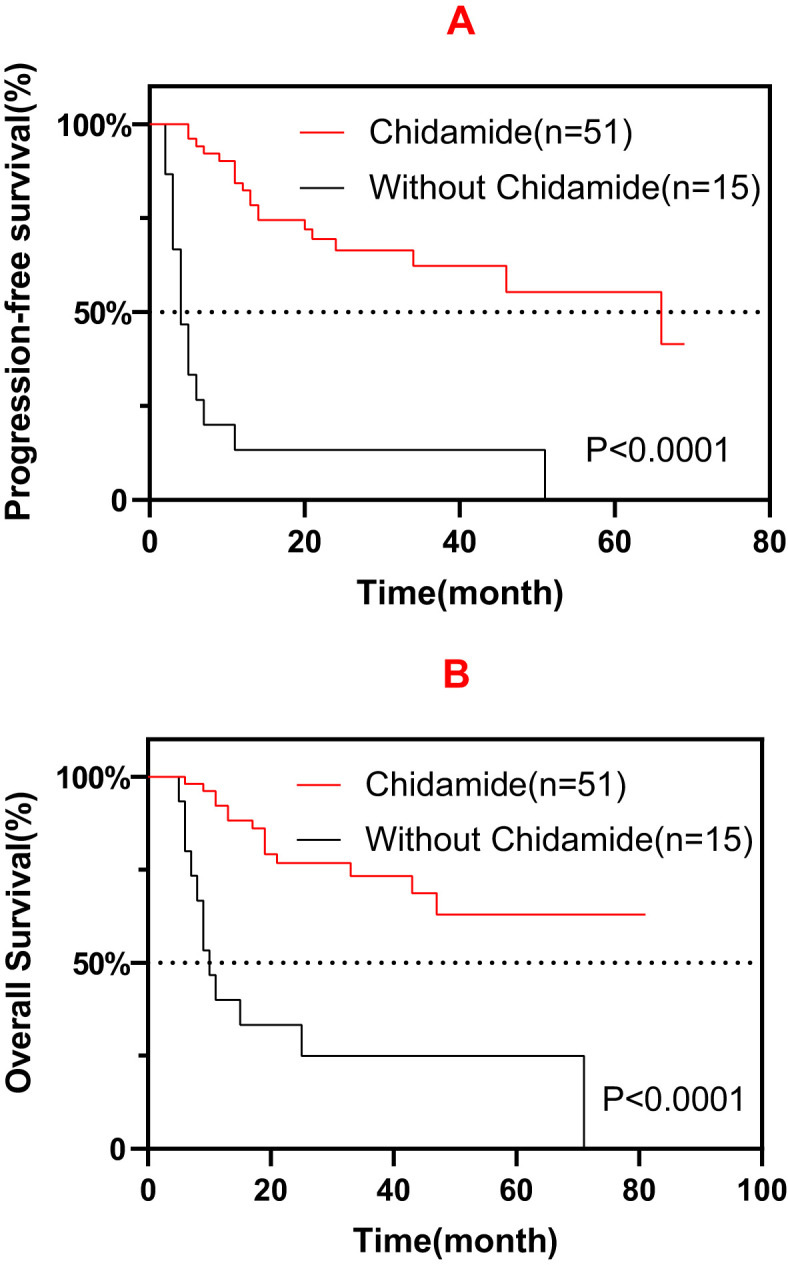
Progression-free survival (PFS) **(A)** and overall survival (OS) **(B)** among patients treated with and without chidamide.

In addition to treated with chidamide, a total of 15 patients diagnosed with ENKTCL (7/15, 46.7%), AITL (4/15, 26.7%), MF (2/15, 13.3%), PTCL-NOS (1/15, 6.7%), and MEITL (1/15, 6.7%) were not treated with chidamide. The patients treated with chidamide had favorable survival compared with patients who did not receive chidamide treatment (**Figures 4A, B**). However, these 15 patients had a much lower response rate to the first-line or salvage treatment (Progressive disease: 14/15, 93.3%).

There were 44 (all grade, 86.3%) patients who had chidamide-related AEs, which were mainly hematological and mild events. There was no treatment-related death recorded. The most common toxicities were neutropenia (all grade, 62.7%), thrombocytopenia (all grade, 49.0%), anemia (all grade, 27.5%), and aminotransferase abnormality (all grade, 17.6%). Of the whole population, severe hematological AEs (grade ≥3) occurred in 11 (21.6%) patients, but severe non-hematological AEs were not recorded (**Table 2**).

**Table 2:** Treatment-related adverse events during Chidamide maintenance therapy.

Adverse event	Any grade, *N* (%)	Grade ≥3, *N* (%)
Neutropenia	32 (62.7)	5 (9.8)
Thrombocytopenia	25 (49.0)	6 (11.8)
Anemia	14 (27.5)	2 (3.9)
Aminotransferase abnormality	9 (17.6)	0 (0)
Renal dysfunction	1 (2.0%)	0 (0)

## Discussion

T- and NK-cell lymphomas are clinically aggressive diseases with a marked heterogeneity among subtypes; however, the treatment options for these diseases are limited due to the large inadequacy of randomized trials and solid observational investigations. HDAC inhibitors appear promising for improving patient outcomes and preventing disease relapse.

In this study, the efficacy of various chidamide-containing regimens was explored as the maintenance treatment for TNKLs. Before the introduction of chidamide in clinical practice, the median survival following salvage therapy for primary refractory TNKLs, such as PTCLs, was 5.8–9.1 months, which is inferior compared with the 24.5 months in patients who received salvage therapy in this study (5, 13). Several studies have demonstrated a prolonged median survival in patients who received high-dose chemotherapy (HDT) followed by autologous stem cell transplantation (ASCT), from 22.9 months to not reached (14, 15). In this study, maintenance therapy with chidamide also demonstrated similar survival benefits (29 months) for patients unsuited for HDT-ASCT. These results could represent an economically applicable alternative therapy for patients with TNKLs instead of tolerating HDT-ASCT and the much higher costs that follow. Furthermore, ENKTCL only accounted for a small proportion of enrolled patients in these studies; moreover, there is still considerable controversy with regard to the application of HDT-ASCT in patients with ENKTCL. The higher incidence of ENKTCL in this study undoubtedly influenced the result of chidamide being a good treatment alternative for patients with ENKTCL. Although the patients treated with chidamide had favorable survival compared with those who did not receive chidamide (**Figures 4A, B**), the much higher rates of CR/PR in the chidamide group might have compromised the survival analysis. Further investigation is warranted to address this issue.

Among patients with PTCL, approximately 70% would eventually experience relapse or refractory disease. Novel drugs and regimens are being explored to address such difficulty. Pralatrexate was the first drug approved for patients with relapsed/refractory PTCLs based on the PROPEL study, but the relatively low objective response rate (ORR) (29%) undermined its clinical application (16). Brentuximab vedotin received approval for the treatment of ALCL or other CD30-expressing PTCLs due to its good efficacy based on the ECHELON-2 study; however, patients with ENKTCL were not enrolled (17). ICIs appear promising for the treatment of ENKTCL, particularly in combination with chidamide, which resulted in 59.5% ORR and prolonged OS (32.9 months) (18). In recent years, HDAC inhibitors have been extensively discovered in hematological malignancies, and the data on PTCLs are encouraging. A preclinical study found that chidamide is synergistic to enhancing the T-cell chemokine expression, increasing the CD8 T-cell infiltration via histone modification (19). Chidamide, as an immunity enhancer, might explain the long-term DOM in this study (median DOM = 14 months). Different from its counterpart, romidepsin, whose approval for the indication of PTCLs was withdrawn due to the high incidence of treatment-related AEs, chidamide showed tolerable AEs, and no treatment-related death was recorded.

Patients who experienced CR after induction therapy had favorable prognosis compared with non-CR patients, but a similar result was not observed in the salvage treatment group, which could be due to the low number of patients and the various treatment lines in these patients. It is worth noting that EI with chidamide did not improve patient outcomes (PFS: *p* = 0.772; OS: *p* = 0.973). Furthermore, 31.2% (10/32) of CR patients *vs*. 56.3% (9/16) of PR patients *vs*. 100% (3/3) of SD patients had chidamide combined induction therapy, possibly indicating that chidamide failed to facilitate induction therapy. Further investigation is warranted to address this issue.

There are several limitations to this study. Firstly, this is a single-center, retrospective, and observational study that constantly faced both selection bias and information bias. Secondly, the low number of enrolled patients and the different subtypes included might have compromised the survival analysis. Thirdly, the treatment options in the salvage setting largely varied among subtypes. Lastly, variant analysis of the chidamide-containing regimens did not proceed according to the PTCL subtypes and treatment lines, with selection bias being inevitable. These limitations could have undermined the integrity of the results of the present study, thus the need for improvement in further research.

In conclusion, chidamide-containing maintenance therapy after induction treatment showed promise and was well tolerated in patients with TNKLs. Further randomized clinical trials that focus on chidamide-containing regimens as a maintenance therapy for TNKLs are needed.

## Data Availability

The raw data supporting the conclusions of this article will be made available by the authors, without undue reservation.
